# Enhanced lightweight and compromised-resilient image encryption for resource constrained environments

**DOI:** 10.1371/journal.pone.0320046

**Published:** 2025-03-26

**Authors:** Abid Mehmood, Abdul Nasir Khan, Iynkaran Natgunanathan, Arslan Shafique, Iftikhar Ahmed Khan, Atta ur Rehman Khan

**Affiliations:** 1 Abu Dhabi University, Abu Dhabi, UAE; 2 COMSATS University Islamabad, Abbottabad Campus, Islamabad, Pakistan; 3 Deakin University, Burwood, Australia; 4 School of Biomedical Engineering, University of Glasgow, Glasgow, United Kingdom; 5 Department of CS & IT, University of Lahore, Lahore, Pakistan; 6 College of Engineering and IT, Ajman University, Ajman, UAE; Civil Aviation University of China, CHINA

## Abstract

The Internet is experiencing a significant increase in multimedia traffic volume, highlighting the growing importance of managing and securing multimedia content efficiently. Classical or traditional security solutions are suitable for those applications that have sufficient computing resources. However, the rise of IoTs and its applications opens new directions for researchers to provide lightweight security solutions. Many IoT applications send critical image data over the Internet, which requires adequate protection. Traditional security solutions are not suitable due to the resource-constrained nature of the environments. An effective security solution is necessary for such environments that balance lightweight design with strong security measures. Current research efforts in this area lack the ability to provide both secure and lightweight properties simultaneously. Therefore, a robust and lightweight cryptosystem is needed to secure the sensitive information of digital images. This research addresses the existing gap by proposing a lightweight and robust cryptographic system that encrypts digital data in less processing time without compromising security. The proposed image encryption technique is evaluated using security and performance measures, such as cipher processing time, histogram analysis, entropy, correlation, mean square error and sensitivity analysis. Moreover, the comprehensive analysis reveals the proposed image encryption technique effectively and collectively meets all the security and performance requirements compared to existing state-of-the-art lightweight image encryption techniques.

## 1. Introduction

IoT applications, such as industrial automation, smartphones, smart transportation, healthcare systems, and environmental monitoring significantly impact our daily lives. These technologies improve productivity, safety, and flexibility of work thus impacting human life positively. Various IoTs applications exchange image data for comprehensive surveillance and analytics, such as the Internet of Battlefield Things (IoBTs) [1, 2], Internet of Drones (IoDs) [3], security surveillance [4], traffic management [5, 6], smart agriculture [7], and healthcare monitoring [8]. Some of the applications mentioned above transmit image data containing highly sensitive information. The loss of such data could lead to financial losses, territorial breaches, or even fatalities. However, IoT environments face specific limitations, including constrained processing power, limited memory, and battery capacity, which create challenges for implementing robust security. Typical security methods are often computationally intensive and, therefore, less suitable for IoT devices [9]. Such devices are deployed in hostile or remote environments, where traditional methods may be impractical. Therefore, a robust cryptosystem is required for such resource-constrained environments to ensure a high level of security [10]. Despite these limitations, IoT devices often handle sensitive data, especially images, requiring advanced security that balances energy efficiency and processing power. Traditional or classical cryptographic algorithms demand high energy and computational resources, which shows there is a pressing need for lightweight encryption solutions that align with IoT device limitations.

Different images vary in size and information content, presenting challenges for encryption within such constrained environments [11]. Key challenges include:

- **Latency:** Real-time applications, such as drone applications, and remote monitoring, require low-latency encryption frameworks to ensure timely data transmission in less processing time.
- **Computational overhead:** Existing encryption systems, such as AES and RSA are computationally expensive due to the involvement of several rounds during encryption and decryption. This property makes them unsuitable for IoT devices.
- **Energy consumption:** IoT devices are often equipped with limited battery capacity. Therefore, such devices need energy-efficient encryption algorithms to carry on their operational life.
- **Memory usage:** Limited memory resources restrict the real-time implementation of conventional cryptographic algorithms. This algorithm requires a large memory for key storage and data processing.

Several lightweight encryption techniques have been proposed to meet the unique needs of IoT environments [12, 13]. The simplified versions of existing proposed image encryption algorithms reduce computational overhead, but they may also compromise security. Thus, the existing lightweight encryption frameworks that are designed to minimize energy and computational demands are insufficient for protecting sensitive image data. Some schemes, like lightweight block ciphers [14, 15] address computational complexity but lack robust security for highly sensitive data. Other methods, such as chaotic encryption systems [16, 17], leverage the properties of chaotic maps, such as sensitivity to initial conditions, which can enhance the data security and resilience against certain attacks. However, these methods face practical challenges. For instance, chaotic encryption systems frequently require precise initial parameters and computationally complex transformations, which may be difficult to maintain on resource-constrained IoT devices like sensors or portable monitoring systems. As a result, these systems may struggle to deliver an ideal balance between security and performance in terms of computational time. This trade-off limits their usability in real-time IoT applications, where both security and operational efficiency are essential. Considering the dual need for lightweight yet robust encryption in IoT, a significant gap remains in existing lightweight encryption schemes. Many existing studies focus on either reducing computational load or only enhancing cryptographic strength, but they often fail to offer a holistic cybersecurity solution suitable to IoT environments [18, 19]. For example, some lightweight encryption techniques, such as simplified block ciphers [20], prioritize minimal energy consumption but may sacrifice security resilience. On the other hand, more secure frameworks, such as advanced cryptographic algorithms [21], typically require intensive processing, which can lead to high latency and reduced battery life on IoT devices. This also makes them unsuitable for real-time applications. To bridge this gap, the proposed research presents a new image encryption scheme that is designed for resource-limited IoT deployments. Unlike traditional encryption methods, which may lack either sufficient security strength or processing efficiency, the proposed approach combines a unique lightweight cryptographic framework that optimally balances these needs. Moreover, the proposed framework significantly reduces power consumption to make it feasible for IoT devices. At the same time, the proposed system incorporates adaptive security mechanisms which are capable of resisting cyberattacks.

### 1.1. Organization of the Paper

Subsequent sections of this paper include: Section 2 summarizes the existing image encryption schemes and identifies their vulnerabilities. Section 3 is devoted to the detailed explanation of the proposed lightweight and compromise-resilient image encryption scheme. Section 4 describes the experimental setup, evaluates the proposed scheme based on performance and security parameters, and compares it with the existing state-of-the-art solutions. Finally, the conclusion is presented in Section 5.

## 2. Literature review

Over the past few years, numerous lightweight image encryption methods have been proposed to encrypt plaintext images with low-latency [22–24]. Although these techniques offer the advantage of reduced processing time, they can compromise security due to their reliance on fewer cryptographic operations, which may weaken overall security. The pros and cons of the most recent image encryption schemes are presented below:

In [25], authors presented a new one-dimensional Piece-Wise Quadratic Polynomial Chaotic Map (PWQPCM) to improve the chaotic properties of the system model, demonstrating superior dynamic performance. The authors also proposed an image encryption algorithm using PWQPCM, integrating pixel segmentation [26], S-box substitution [27], and diffusion encryption [28] that provides advantages such as data loss resilience, low computational overhead, and adjustable security strength. However, vulnerabilities arise from parameter selection weaknesses and initial condition sensitivity of the chaotic map that compromises security under several cyberattacks. In [29], authors use topological conjugate theory for generating a cluster of 1D quadratic chaotic maps, featuring three adjustable parameters that greatly expand the parameter space unlikely traditional 1D chaotic maps. The theoretical foundation demonstrates the chaotic nature of these maps through their topological conjugation with a logistic chaotic map, validated by numerical simulations showing robust chaotic behavior and confirming theoretical analyses. Despite these promising characteristics, their proposed image encryption algorithm, relying only on the properties of chaotic maps for security, fails multiple NIST randomness tests such as Frequency, BlockFrequency, CumulativeSums, LongRuns, and Rank. This suggests potential vulnerabilities in the cryptographic strength of the encryption scheme. In [30], authors proposed an image encryption algorithm by combining three modified 1D chaotic maps to overcome the limitations of existing 1D and multidimensional chaotic maps. Initialization of chaotic maps and diffusion phase employ a key image as both an initializer and a mask via the XOR operator. The algorithm uses ExtraParm from the input plaintext image. Moreover, it consists of both confusion and diffusion operation in its encryption process. Although it passes several standard tests, but it fails various NIST randomness tests, such as BlockFrequency, NonOverlappingTemplate, OverlappingTemplate, Universal, and ApproximateEntropy. This weakness makes them non-resistive against statistical attacks that need further research and improvement.

In [31], the authors propose a protocol called Message Queuing Telemetry Transport (MQTT) [32] for an end-to-end chaotic encryption scheme and the secure transmission of medical images. The method is evaluated using several analyses, such as maximal Lyapunov exponent analysis, NIST SP 800–22, and TestU01 statistical tests. It proved effective against differential attacks, pixel correlation, histogram analysis, entropy assessment, and keyspace attacks. However, it failed several NIST randomness tests, including NonOverlappingTemplate, OverlappingTemplate, Universal, and ApproximateEntropy, indicating potential statistical vulnerabilities. Despite this, the encryption scheme is suitable for edge computing devices and demonstrated high throughput capabilities on Raspberry Pi and desktop PCs. The proposed image encryption scheme of [33] divides images into uniformly sized tiles and generates chaotic sequences for each tile individually. This approach minimizes the computational load by generating sequences for smaller tiles instead of the entire larger image. However, an issue identified with the scheme is the loss of plaintext data due to the truncation of pixel columns and rows at the edges of the ciphertext image. This loss results in the irreversible removal of part of the plaintext data during decryption from the ciphertext image. Moreover, the encryption scheme failed two NIST randomness tests including ApproximateEntropy and Serial. In [34], Kumar *et al.* proposed a lightweight encryption scheme for secure network data transmission, using DNA-based methods and a composite random number generator to encrypt images by sequentially scrambling and diffusing data. The encryption relies on a hybrid PRNG built from a chaotic map and a 256-bit shift register for enhanced security. In [35], Kumar *et al.*, introduced a nonlinear feedback shift register (NLFSR) in Galois configuration for lightweight encryption, applying feedback to each state to enhance pseudorandom number generation. The compact design, validated with NIST tests, is used in a two-phase image encryption algorithm featuring permutation and diffusion. In [36], Kumari *et al.* introduced a lightweight stream cipher that ensures privacy, data integrity, and device integrity through a message authentication code (MAC). The cipher utilizes a nonlinear feedback shift register (NLFSR) and generates the MAC by concatenating a physical unclonable function (PUF) response with the hash of the plaintext, enhancing protection for both data and devices.

In [37], authors proposed an image encryption scheme by incorporating chaos theory. Their method utilizes multiple chaotic maps such as Arnold’s cat map [38] and chaotic gingerbread map. To enhance security through confusion and diffusion purposes, the key generation and pixel manipulation are performed according to the random sequences generated using chaotic maps. However, the robustness of their proposed scheme based on lightweight chaotic maps is compromised by susceptibility to cryptanalytic attacks as it reveals predictable patterns. In [39], authors identified vulnerabilities in the Nested Circular Image Encryption Scheme (NCIES). The NCIES is susceptible to chosen plaintext/ciphertext attacks when implemented with a single round. To address these issues, the authors proposed a new lightweight dynamic key-dependent cipher scheme to enhance the security of digital images. Their proposed encryption scheme aims to enhance the cipher’s resilience against adversarial attacks while optimizing latency and resource utilization. Therefore, it provides a more balanced approach compared to current chaotic image cipher schemes.

In [40], authors introduced a lightweight image encryption technique emphasizing bit-level permutation to enhance security and reduce computational overhead. Their approach incorporates cascade cross-circular diffusion and bit-level confusion to enhance the pixel permutation process. However, the high reliance on bit-level permutation introduces vulnerabilities to cryptanalysis that expose patterns and compromise security. In [41], authors introduced an image encryption scheme by combining Nonlinear Feedback Shift Register (NLFSR) [42] and DNA computation [43]. Their approach begins with image permutation using an NLFSR-generated pseudorandom sequence. Afterward, pixel substitution is performed using DNA computations in cipher block chaining mode. While their technique demonstrated resilience in security and performance analyses against various attacks, vulnerabilities stem from the predictable nature of NLFSR-generated sequences that compromise cryptographic strength if the sequence generation process is weak. In [44], authors implemented a chaotic oscillator based on a second-order differential equation for encrypting plaintext images through confusion and diffusion operations. Their approach incorporates chaotic sequences from this oscillator to scramble image pixels. While chaos-based methods are resilient against chosen plaintext and brute force attacks due to their inherent randomness and uncertainty, challenges arise from the deterministic nature of chaotic systems that lead to predictability under specific conditions.

A summary of the existing encryption framework is provided in Table 1.

**Table 1:** Summary of existing encryption frameworks.

Methodology	Real-World Performance	Robustness Against Attacks	Advantages	Disadvantages	Potential
[25]	Superior dynamic performance	Vulnerable to parameter selection and initial conditions	Robust chaotic behavior	Potential security compromises	Improved parameter management
[29]	Robust chaotic behavior	Fails NIST randomness tests, vulnerable to statistical attacks	Expanded parameter space	Cryptographic weaknesses	Enhanced statistical robustness
[30]	Passes standard tests	Vulnerable to NIST randomness tests, statistical attacks	Utilizes pixel segmentation	Susceptibility to specific attacks	Advanced statistical analysis
[31]	High throughput, edge computing suitability	Fails NIST randomness tests, statistical vulnerabilities	Effective against differential attacks	Statistical test failures	Statistical test improvements
[33]	Lightweight, secure	Fails NIST randomness tests, Data loss due to edge truncation	Reduced computational load	Irreversible plaintext loss	Improved edge handling
[37]	Utilizes chaos theory	Vulnerable to cryptanalytic attacks	Enhanced confusion and diffusion	Predictable patterns	Advanced chaos parameterization
[39]	Balanced security, resource optimization	Vulnerable to chosen plain text/ciphertext attacks	Improved security resilience	Vulnerabilities in single-round NCIES	Enhanced key management
[40]	Reduced computational overhead	Vulnerable to cryptanalysis	Enhanced permutation process	Pattern exposure	Advanced permutation techniques
[41]	Resilient performance	Vulnerable to NLFSR predictability	Combines chaotic and DNA methods	Predictable sequence issues	Enhanced randomness integration
[44]	Confusion and diffusion operations	Deterministic chaos, predictability	Resilient against chosen plaintext attacks	Deterministic behavior	Improved chaos parameter dynamics

### 2.1. Contributions of the paper

**Fig 1:**
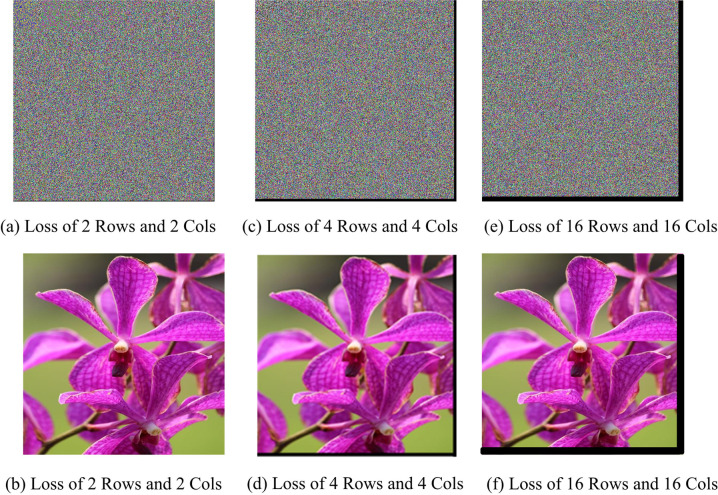
(a) Encrypted Image with tiles 7 ×  7 (b) Decrypted Image with tiles 7 ×  7 (c) Encrypted Image with tiles 17 ×  17 (d) Decrypted Image with tiles 17 ×  17 (e) Encrypted Image with tiles 21 ×  21 (f) Decrypted Image with tiles 21 ×  21.

Section 2 discusses several vulnerabilities in the existing encryption schemes. To address these challenges, the proposed work contributes the following:

- The proposed research introduces a new image encryption system specifically designed for resource-constrained environments, such as IoT applications. This encryption framework effectively balances lightweight design with strong security measures.
- The study identifies a significant gap in existing image encryption schemes discussed in the literature review. Existing cybersecure solutions fail to provide both lightweight and secure encryption simultaneously. The proposed work aims to bridge this gap by proposing a new methodology that does not compromise computational overhead and robust security.
- The proposed image encryption technique extends our previously published image encryption scheme [33], which suffered from the limitation of losing plaintext data due to the truncation of pixel columns and rows at the edges of the ciphertext image, illustrated in Fig 1. This loss entails the irreversible removal of portions of plaintext data during decryption from the ciphertext image. The newly proposed image encryption technique addresses this issue as well.

## 3. Proposed Lightweight Image Encryption in Resource-constrained Environments

**Fig 2:**
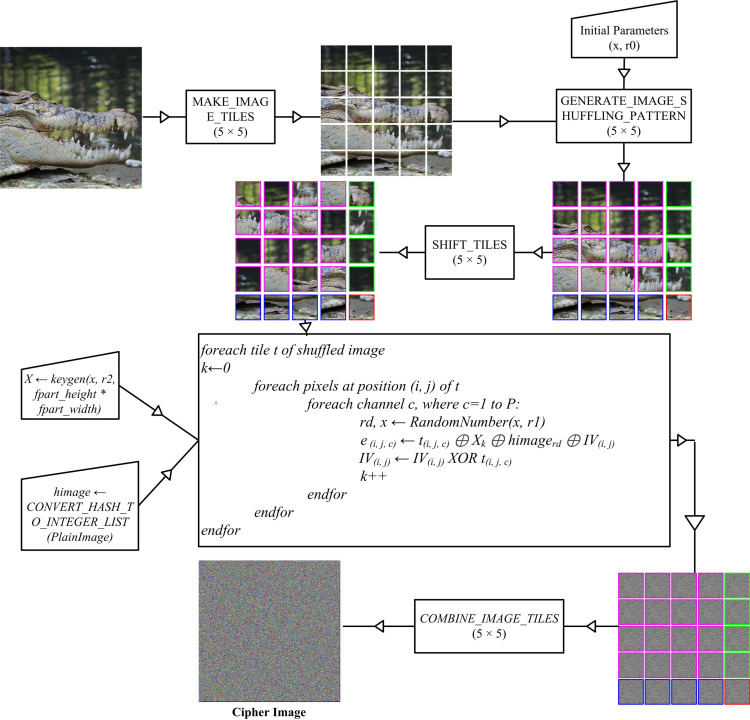
Flow of proposed enhanced lightweight and compromise-resilient image encryption scheme for resource constrained environment.

The proposed image encryption scheme consists of five phases: (a) Make Image Tiles, (b) Generate Image Shuffling Pattern, (c) Shift Tiles, (d) Encrypt Tiles, and (e) Combine Image Tiles. Fig 2 illustrates the flow of the proposed scheme.

The proposed technique removes the limitations of existing lightweight image encryption techniques by dividing the image into a two-dimensional matrix of tiles, each potentially having different dimensions, using the following algorithms.

**Table:** 

***Algorithm 1 MAKE_IMAGE_TILES(image, R, C)*** ***Input*** *: image - the input image* *R - the number of rows* *C - the number of columns* ***Output*** *: image_parts - a list containing the tiled parts of the image* *part_height - height of each part* *part_width - width of each part* *fpart_height - height of the final part* *fpart_width - width of the final part* *num_rows ← R* *num_cols ← C* *height, width ← Length(image) // Get the dimensions of the input image* *part_height ← Floor(height / num_rows) // Calculate the height of each part* *part_width ← Floor(width / num_cols) // Calculate the width of each part* *fpart_height ← part_height + height % num_rows // Calculate the height of the final part* *fpart_width ← part_width + width % num_cols // Calculate the width of the final part* *image_parts ← [] // Initialize an empty list to store the tiled parts of the image* *For r from 0 to num_rows - 1 do* *For c from 0 to num_cols - 1 do* *If r + 1 == num_rows and c + 1 == num_cols then* y_start ← r * part_height y_end ← (r + 1) * part_height + height % num_rows x_start ← c * part_width x_end ← (c + 1) * part_width + width % num_cols *ElseIf r + 1 == num_rows then* y_start ← r * part_height y_end ← (r + 1) * part_height + height % num_rows x_start ← c * part_width x_end ← (c + 1) * part_width *ElseIf c + 1 == num_cols then* y_start ← r * part_height y_end ← (r + 1) * part_height x_start ← c * part_width x_end ← (c + 1) * part_width + width % num_cols *Else* y_start ← r * part_height y_end ← (r + 1) * part_height x_start ← c * part_width x_end ← (c + 1) * part_width *End If* *part ← image[y_start:y_end, x_start:x_end] // Crop the part from the original image* *Append part to image_parts // Add the part to the list of image parts* ***Return image_parts, part_height, part_width, fpart_height, fpart_width*** *// Return the image parts along with their dimensions*

The input to this algorithm is the original image to be encrypted, along with the specified number of rows and columns into which the image will be split. This algorithm creates four different-sized tiles. The dimensions of all the tiles in the last row are equal to *fpart_height* ×  *part_width*, except for the last tile of the row. Similarly, the dimensions of all the tiles in the last column are equal to *part_height* ×  *fpart_width*, except for the last tile of the column. The tile at the intersection of the last row and last column has unique dimensions of *fpart_height* ×  *fpart_width* unmatched by any other tile. The dimensions of all remaining tiles are uniform and equal to *part_height* ×  *part_width*. In Fig 2, each of the four differently sized tiles is visually distinguished by its unique border color. All tiles of an image are appended in a list named *image_parts*.

Subsequently, a tile shuffling pattern is generated utilizing the following algorithm. This algorithm employs initial parameters *(x, r0)* to generate chaotic values of size *R ×  C* using the logistic map. These chaotic values are then utilized to shuffle the list index containing sequential values ranging from 0 to *(R ×  C)-1*. During the generation of the shuffling sequence, it is ensured that tiles of the same size are shuffled exclusively with each other; tiles of different sizes are not shuffled together.

**Table:** 

***Algorithm 2 GENERATE_IMAGE_SHUFFLING_PATTERN(x, r0, R, C)*** ***Input:*** *x - initial value for the shuffling pattern* *r0 - constant value for shuffling* *R - number of rows* *C - number of columns* ***Output:*** *index - a list containing the shuffled indices* *Initialize index as an empty list* *Initialize k as an empty list* *For i from 0 to R×C - 1 do* x ← (r0^2 * (x^2 - 5) * (1 - r0 * (x^2 - 5))) % 1 *Append x to k* *Append i to index* For i from 0 to R*C - 2 do For j from 0 to R*C - 2 do *If (i + 1) % C == 0 then* *If k[i] > k[j] then* If i + C != R*C - 1 then *Swap k[i] and k[i+C]* *Swap index[i] and index[i+C]* *ElseIf (j + 1) % C == 0 then* *If k[i] > k[j] then* If (j + C) != (R*C– 1) then *Swap k[j] and k[j+C]* *Swap index[j] and index[j+C]* ElseIf i >= (R-1) * C then *If k[i] > k[j] then* If i + 1 != R*C - 1 then *Swap k[i] and k[i+1]* *Swap index[i] and index[i+1]* ElseIf j >= (R-1) * C then *If k[i] > k[j] then* If j + 1 != R*C - 1 then *Swap k[j] and k[j+1]* *Swap index[j] and index[j+1]* *Else* *If k[i] > k[j] then* *Swap k[i] and k[j]* *Swap index[i] and index[j]* ***Return index***

Once the shuffling index is generated, the tiles are shuffled using the algorithm described below. The shuffling process is depicted in Fig 2. All tiles are shuffled except for the last one, which is situated at the intersection of the last row and last column, as there is no other tile available with the same size.

**Table:** 

***Algorithm 3 SHIFT_TILES(tiles, R, C, x, r)*** ***Input:*** *tiles - a list containing the tiles of the image* *R - the number of rows in the grid of tiles* *C - the number of columns in the grid of tiles* *x - initial value for the shuffling pattern* *r - constant value for shuffling* ***Output*** *: stiles - a list containing the shifted tiles* Initialize stiles as a list of None with length R * C *index ← GENERATE_IMAGE_SHUFFLING_PATTERN(x, r, R, C)* For i from 0 to (R * C– 1) do *stiles[i] ← tiles[index[i]]* ***Return stiles***

After compiling the tile shuffling, every pixel of each tile is encrypted. Prior to encrypting the tiles, a key sequence named X is generated using the procedure explained in the following algorithm.

**Table:** 

***Algorithm 4 Generate_Key(x, r2, rows, cols)*** ***Input*** *: (x, r2) – initial parameters* *rows - the number of rows in the last tile* *cols - the number of columns in the last tile* ***Output*** *: X – key of size rows × cols* *Initialize key as a list of None with length rows × cols* *For i form 1 to range(rows × cols) do* x← r2*x*(1-x) key.append(int(x*pow(10,12))%256) *Return* ***key***

The sequence X’s size matches fparthight ×  fpartwidth, representing the maximum tile dimensions, making it ideal for encrypting each image tile. Additionally, the encryption process requires the hash value of the original image. This value is derived through the standard SHA-512 algorithm and subsequently transformed into an array of integers, containing 64 values, as per the following procedure:

**Table:** 

***Algorithm 5 CONVERT_HASH_TO_INTEGER_LIST(img, str1)*** ***Input*** *: img - the image to be hashed* *str1 - additional string for hashing* ***Output*** *: hkey - a list of integers derived from the hash* *Calculate the SHA3-512 hash of the concatenation of str(img) and str1 encoded in UTF-16* *Convert the hexadecimal hash digest to a list of integers* *Return* ***hkey***

The third requisite for encrypting the image is an IV (Initialization Vector) array, initialized with zeros and having dimensions of fpart_height ×  fpart_width. Initially set to zero, the IV progressively stores the XORed value of preceding tiles, which is then utilized in the encryption of the current tile to introduce randomness in the encrypted image. The encryption procedure of the proposed technique is explained in the following algorithm.

**Table:** 

***Algorithm 6 ENCRYPT_IMAGE(img, hkey, himage, R, C, r0, r1, r2, x)*** ***Input*** *: img - the original image to be encrypted* *hkey - encryption key* *himage - image for XOR operation* *R - number of rows for tiling* *C - number of columns for tiling* *r0, r1, r2 - parameters for encryption* *x - initial value for the shuffling pattern* ***Output*** *: ceimg - the encrypted image* *tiles, part_height, part_width, fpart_height, fpart_width ← MAKE_IMAGE_TILES(img, R, C)* X ← keygen(x, r2, fpart_height * fpart_width) *stiles ← SHIFT_TILES(tiles, R, C, x, r0)* *etiles ← []* IV ←array of zeros with shape of fpart_height * fpart_width *For each tile stile in stiles do* *trows ← height of stile* *tcols ← width of stile* *img1 ← array of zeros with shape of stile* *k ← 0* *For i from 0 to (trows – 1) do* *For j from 0 to (tcols – 1) do* *rd, x ← RandomNumber(x, r1)* *img1[i, j] ← stile[i, j] XOR X[k] XOR himage* *[31] XOR IV[i, j]* *IV[i, j] ← IV[i, j] XOR stile[i, j]* *k ← k + 1* *Append img1 to etiles* *ceimg ← COMBINE_IMAGE_TILES(etiles, R, C)* *Return* ***ceimg***

The single pixel of the encrypted image is dependent on the current pixels value of the original image xored with the key value at k^th^ position of X, rd^th^ value selected from the integer array of hash value of the original image, and IV which contains the xored information of previous tiles in original form. The process of selecting the rd^th^ value is shown in the following algorithm.

**Table:** 

***Algorithm 7 RandomNumber (x, r1)*** ***Input*** *: (x, r1) – initial parameters* ***Output*** *: rnd - the randomly generated* x=r*x*(1-x) rnd=(math.floor(x*math.pow(7,13)-1))%64 ***Return*** *rnd,x*

Once the encryption process for each tile is finished, all tiles are merged using the following algorithm to create the cipher image.

**Table:** 

***Algorithm 8 COMBINE_IMAGE_TILES(tiles, rows, cols)*** ***Input*** *: tiles - a list containing the tiles of the image* *rows - the number of rows in the original image* *cols - the number of columns in the original image* ***Output*** *: reconstructed_im - the reconstructed image* *Initialize reconstructed_im as None* *// Iterate through rows* *For i from 0 to rows - 1 do* *// Initialize row image* *Initialize row_im as None* *// Iterate through columns* *For j from 0 to cols - 1 do* *// Calculate tile index* tile_index ← i * cols + j *// Check if tile index is within range* *If tile_index < Length(tiles) then* *// Get the current tile* *tile ← tiles[tile_index]* *// Update row image* *If row_im is None then* *row_im ← tile* *Else* *row_im ← Concatenate(row_im, tile) along axis 1* *End If* *End For* *// Update reconstructed image* *If reconstructed_im is None then* *reconstructed_im ← row_im* *Else* *reconstructed_im ← Concatenate(reconstructed_im, row_im) along axis 0* *End For* ***Return reconstructed_im***

The encryption process utilizes two key elements: the initial value of parameter x, employed in generating chaotic values, and the hash value of the original image. The remaining initial parameters (r0, r1, r2) are derived from the hash value of the original image. Below is the algorithm depicting the initialization parameters utilized in the proposed technique:

**Table:** 

***Algorithm 9 PARAMETER_INTIALIZATION()*** *img ← Read image from file* *X ← Empty list to store X values* *rows ← Number of rows in img* *cols ← Number of columns in img* *R ← Total Rows of Tiles* *C ← Total Columns of Tiles* *himage ← CONVERT_HASH_TO_INTEGER_LIST (img, "")* *var0 ← HKEY_SEQUENCE (himage, 0, 4)* *var1 ← HKEY_SEQUENCE (himage, 1, 4)* *var2 ← HKEY_SEQUENCE (himage, 1, 1)* *r0 ← CONVERT_TO_FLOAT (var0)* *r1 ← CONVERT_TO_FLOAT (var1)* *r2 ← CONVERT_TO_FLOAT (var2)*

The HKey_SEQUENCE is employed to generate an integer value by adding the integer hash value of the original image. Subsequently, this value serves as a seed to produce a random floating-point number falling between 3.57 and 4.0, which is then utilized as initialization parameters. The procedure is illustrated in the following algorithms:

**Table:** 

***Algorithm 10 HKEY_SEQUENCE(hkey, start, jump)*** ***Input*** *: hkey - the sequence of keys* *start - the starting index for the sequence* *jump - the increment value for the sequence* ***Output*** *: sum - the sum of the elements in the sequence* *Initialize sum as 0* *For i from start to length of hkey - 1, with step size jump do* *sum ← sum + hkey[i]* ***Return*** *sum*

**Table:** 

***Algorithm 11 CONVERT_TO_FLOAT(seed)*** ***Input*** *: seed - the seed value for the random number generator* ***Output*** *: random_float - a randomly generated floating-point number* *Set the random seed to seed* *Generate a random floating-point number between 3.57 and 4.0* ***Return*** *random_float*

The cipher image, accompanied by the key value x and the hash of the original image, is transmitted to the receiver for decryption. Since the key value changes for every image, it can be utilized as a temporary session key and transmitted in the form of an envelope using public key cryptography. Upon receiving the envelope containing the encrypted image and the keys of the encrypted image, encrypted with the private key of the receiver, the receiver decrypts the keys and generates the sequence (r0, r1, r2) and X using the same procedure discussed previously. The decryption process of the image is outlined in the following algorithm:

**Table:** 

***Algorithm 12 DECRYPT_IMAGE(eimg, hkey, himage, R, C, r0, r1, r2, x)*** ***Input*** *: eimg - the encrypted image to be decrypted* *hkey - encryption key* *himage - image for XOR operation* *R - number of rows for tiling* *C - number of columns for tiling* *r0, r1, r2 - parameters for decryption* *x - initial value for the shuffling pattern* ***Output*** *: pimg - the decrypted image* *etiles, part_height, part_width, fpart_height, fpart_width ← MAKE_IMAGE_TILES(eimg, R, C)* X ← keygen(x, r2, fpart_height * fpart_width) *tiles ← []* IV ←array of zeros with shape of fpart_height * fpart_width *For each tile etile in etiles do* *trows ← height of etile* *tcols ← width of etile* *img1 ← array of zeros with shape of etile* *k ← 0*
*For i from 0 to trows - 1 do* *For j from 0 to tcols - 1 do* *rd, x ← RandomNumber(x, r1)* *img1[i, j] ← etile[i, j] XOR X[k] XOR himage* *[31] XOR IV[i, j]* *IV[i, j] ← IV[i, j] XOR stile[i, j]* *k ← k + 1* *Append img1 to tiles* *invtiles ← INV_SHIFT_TILES(tiles, R, C, x, r0)* *pimg ← COMBINE_IMAGE_TILES(invtiles, R, C)* ***Return*** *pimg*

In the same manner, the encrypted image is partitioned into multiple tiles. Subsequently, each pixel is decrypted by performing the XOR operation on the currently encrypted pixels, the k^th^ value of X, the rd^th^ value selected from the integer array of hash value of the original image, and IV which contains the XORed information of the previous tiles in plain form. Afterwards, the decrypted tiles are reshuffled back to their original positions using the following algorithm:

**Table:** 

***Algorithm 13 INV_SHIFT_TILES(stiles, R, C, x, r)*** ***Input*** *: stiles - a list containing the shifted tiles* *R - the number of rows in the grid of tiles* *C - the number of columns in the grid of tiles* *x - initial value for the shuffling pattern* *r - constant value for shuffling* ***Output*** *: temp - a list containing the inverted shifted tiles* Initialize temp ← None with length R * C *index ← GenerateImageShufflingPattern(x, r, R, C)* For i from 0 to R * C - 1 do *temp[index[i]] ← stiles[i]* ***Return*** *tem*

**Fig 3:**
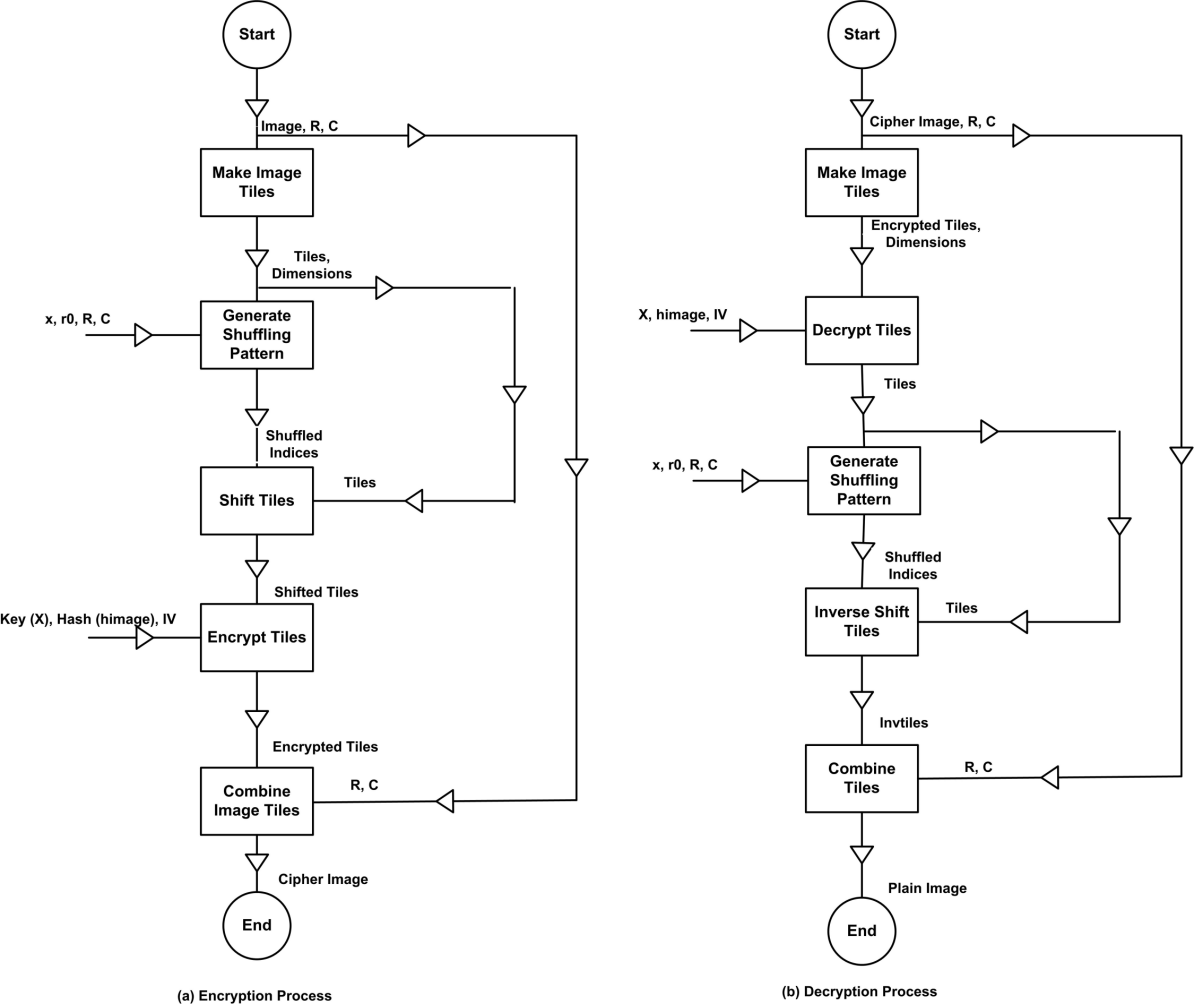
Encryption and Decryption Flow of the proposed image encryption technique.

Thereafter, the decrypted tiles are merged again using the same process explained earlier to form the decrypted image. The flow of the encryption and decryption processes are shown Fig 3.

In designing this scheme, particular attention is paid to minimizing the number of values used as secret keys to alleviate the burden of securing and distributing unnecessary keys. The proposed image encryption technique relies solely on two values that require protection: (a) a chaotic sequence X and (b) the 512-bit hash of the original image. The remaining secret values are generated using the algorithms mentioned above. Secondly, this scheme generates the key X equal to the tile size of fpartheight ×  fpartwidth using chaotic maps. This approach contrasts with existing schemes, which typically generate more than one key sequence, with many of them being equal to the width and height of the image. Therefore, dividing the image into multiple tiles and generating a key equal to the tile size enhances performance in terms of space and time during the key generation process. Thirdly, to encrypt the n^th^ tile, it undergoes XOR operations with the secret key X, the hash value of the original image, and IV, which results from the XOR operation of the previous n-1 tiles. The involvement of IV generates a highly randomized cipher image that is difficult to crack. When the cipher image encrypted with the proposed scheme is subjected to the NIST randomness test suite, it passes all the randomness tests mentioned in the test list. The results are shown in Table 2.

**Table 2:** NIST randomness tests result conducted on an encrypted image of a car.

	P-VALUE	PROPORTION	Status
**Frequency**	0.739918	10/10	Pass
**BlockFrequency**	0.911413	10/10	Pass
**CumulativeSums**	0.350485	10/10	Pass
**Runs**	0.213309	10/10	Pass
**LongestRun**	0.534146	10/10	Pass
**Rank**	0.534146	10/10	Pass
**FFT**	0.911413	10/10	Pass
**NonOverlappingTemplate**	0.066882	10/10	Pass
**OverlappingTemplate**	0.534146	10/10	Pass
**Universal**	0.350485	10/10	Pass
**ApproximateEntropy**	0.911413	10/10	Pass
**RandomExcursions**	----	4/4	Pass
**RandomExcursionsVariant**	----	4/4	Pass
**Serial**	0.350485	10/10	Pass
**LinearComplexity**	0.534146	10/10	Pass

**Fig 4:**
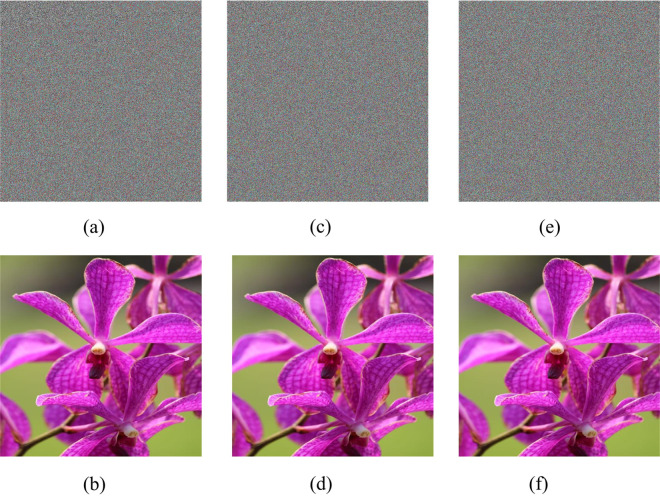
(a) Encrypted Image with tiles 7 ×  7 (b) Decrypted Image with tiles 7 ×  7 (c) Encrypted Image with tiles 17 ×  17 (d) Decrypted Image with tiles 17 ×  17 (e) Encrypted Image with tiles 21 ×  21 (f) Decrypted Image with tiles 21 ×  21.

Lastly, the proposed technique divides the image into multiple tiles, which may not necessarily be equal in size. This option facilitates encrypting the entire image and decrypting it without losing a single bit, unlike existing image encryption schemes. The outcomes of encryption and decryption, employing varying rows and columns, are depicted in Fig 4.

## 4. Result and Discussion

We conducted an implementation and comparison of the proposed image encryption scheme with state-of-the-art lightweight image encryption schemes. The experiments were performed on a system having Intel(R) Core(TM) i7-10870H CPU @ 2.20GHz and 16GB of RAM. Our evaluation of the proposed image encryption technique involved a comprehensive comparison with existing schemes across various metrics, including encryption/decryption time, histogram analysis to examine pixel intensity distribution in both encrypted and original images, entropy for randomness assessment, correlation to analyze the relationship between adjacent pixels in plain and encrypted images, Mean Square Error (MSE) for quantifying differences between the original and encrypted images, and sensitivity analysis to evaluate the avalanche effect. We utilized eight images of varying dimensions and sizes for comparison, sourced from the USC-SIPI (https://sipi.usc.edu/database/) and CVG-UGR (https://ccia.ugr.es/cvg/dbimagenes/) image databases. Further information regarding the images used in the experiment is provided in Table 3.

**Table 3:** Image dataset of from USC-SIPI and CVG-UGR.

Name	Size	Bit Depth	Dimension
Car	4.88MB	24 bits	1600 × 1067
Oakland	3MB	24 bits	1024 × 1024
Airport	3MB	24 bits	1024 × 1024
Baboon	768KB	24 bits	512 × 512
Peppers	768KB	24 bits	512 × 512
Airplane(F-16)	768KB	24 bits	512 × 512
House	768KB	24 bits	512 × 512
Jelly Beans	256 KB	24 bits	256 × 256

Since the proposed scheme extends our previously published research, we have used the same latest schemes for comparison as those employed in [33], which include [25,29,30], and [31].

### 4.1. Encryption/Decryption Time

**Fig 5:**
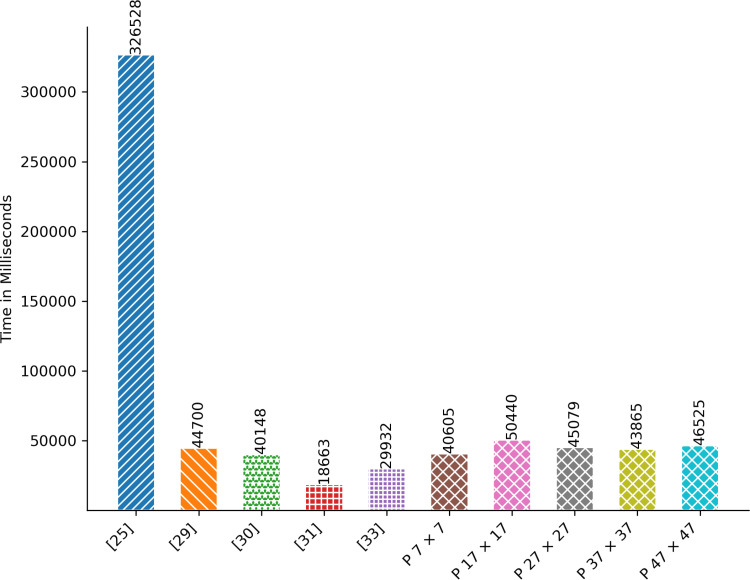
Aggregated Encryption Time in Milliseconds.

**Fig 6:**
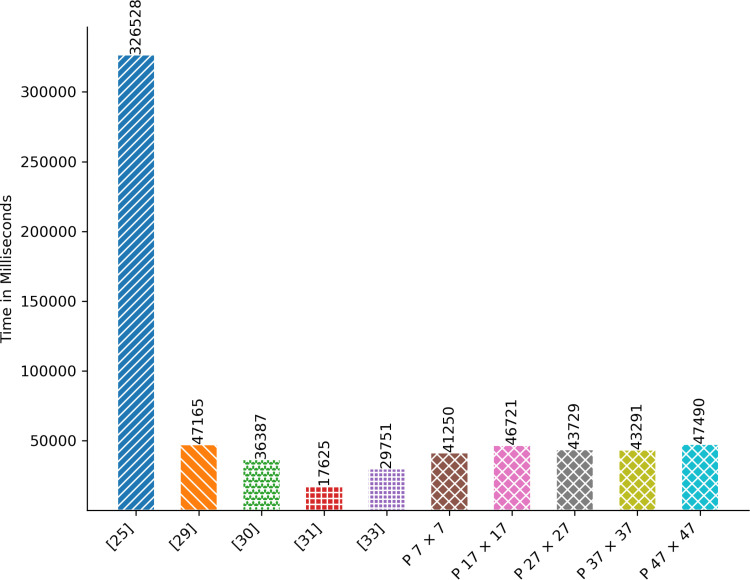
Aggregated Decryption Time in Milliseconds.

The time measured in milliseconds denotes the duration required for encrypting or decrypting all images specified in Table 3. Figs 5 and 6 depict the performance of the proposed technique in terms of encryption/decryption time. It surpasses [25,29] when employing a tile size of 7 by 7. Moreover, the encryption/decryption time of the proposed technique closely aligns with that of [30]. Nonetheless, the encryption/decryption time observed in [31,33] outperforms that of the proposed technique.

**Fig 7:**
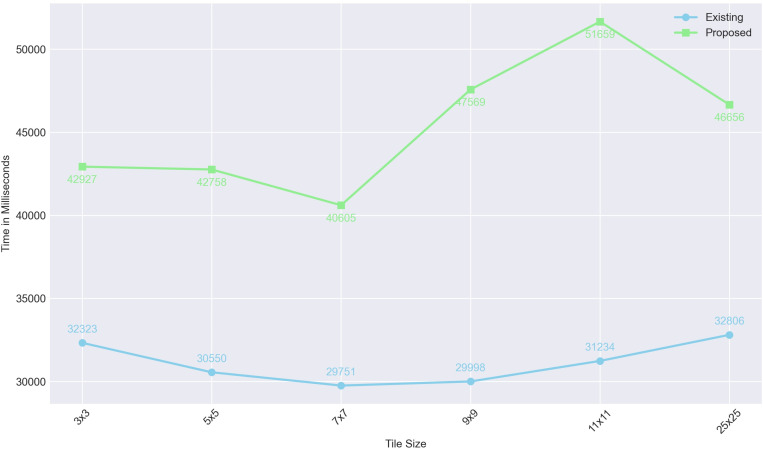
Encryption Time with different tile sizes.

The technique presented in [33] also divides the image into multiple tiles before executing encryption and decryption operations. Fig 7 demonstrates the performance of [33] compared to the proposed technique across various tile sizes. The results indicate that the scheme presented in [33] outperforms the proposed technique in encrypting the dataset used in the experiment.

While the proposed technique may require slightly more time for encryption/decryption compared to the existing schemes, it is noteworthy that most of the existing schemes fail many NIST randomness tests. In contrast, our proposed scheme successfully passes all the NIST randomness tests.

### 4.2. Histogram Analysis

**Fig 8:**
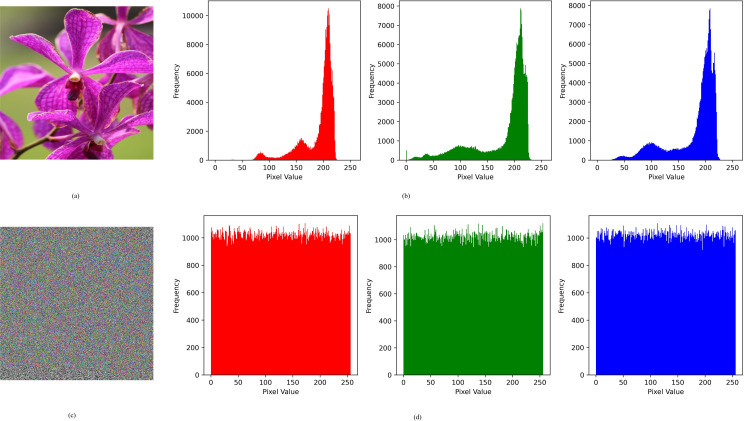
Histogram Analysis of Plain and Encrypted Image.

This analysis helps in examining the impact of the encryption process, encompassing the distribution of pixel intensities throughout the image. The plain image exhibits a uniform distribution of pixel intensity, whereas the encrypted image reveals a distinct pattern stemming from alterations in pixel intensity distribution caused by encryption. Fig 8 demonstrates that the proposed image encryption scheme substantially modifies the distribution of pixel intensities across the encrypted image when compared to the plain image. This alteration is crucial for upholding the security of information contained within the image.

### 4.3. Entropy analysis

**Fig 9:**
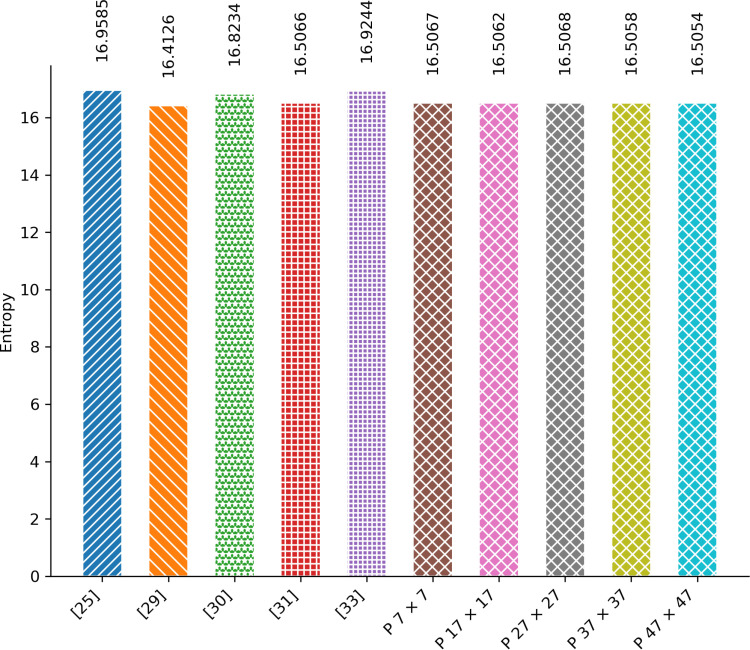
Entropy Analysis.

Entropy serves as a metric for quantifying the randomness or unpredictability of pixel values in an image. If there is minimal variation in the intensity of image pixels, the entropy value will be low; conversely, if there is significant variation in pixel intensity, the entropy will be high. Plain images typically exhibit lower entropy, whereas encrypted images tend to have higher entropy due to the encryption process enhancing the variation in pixel intensity. Therefore, a higher entropy value in an encryption algorithm signifies the introduction of greater randomness in the encrypted image, which is a desirable feature. Fig 9 illustrates a comparison of the entropy values between the proposed scheme and existing image encryption schemes.

The graph illustrates the average entropy observed in the encrypted images across the dataset. Notably, the average entropy value among all plain images in the dataset stands at 14.33. It is evident from the graph that all schemes introduce nearly the same levels of entropy into the encrypted images. The entropy of [25,30], and [33] slightly exceeds the proposed scheme, while the entropy of [29] and [31] falls slightly below the proposed schemes.

### 4.4. Correlation analysis

**Fig 10:**
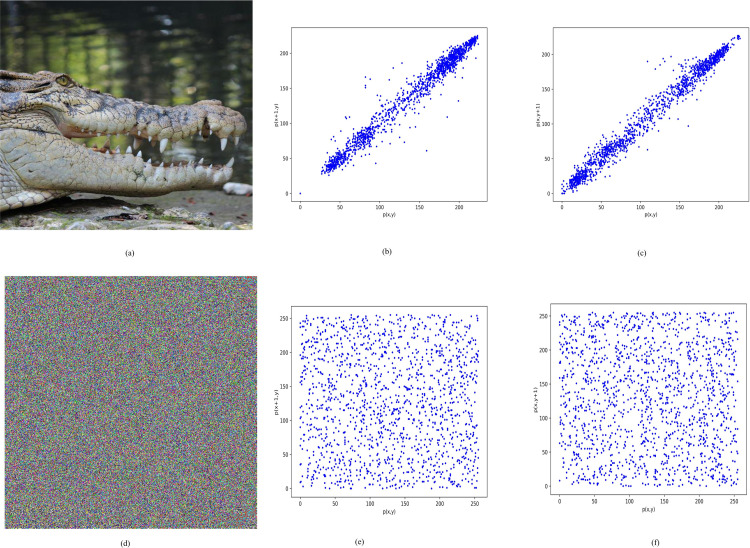
The correlation between the adjacent rows and columns and (a) plain image (b) relationship between the adjacent columns in the original image (c) relationship between the adjacent rows in the original image (d) encrypted Image (e) relationship between the adjacent columns in the encrypted image (f) relationship between the adjacent rows in the encrypted image.

The correlation analysis provides information about the similarity or relationship between two images or two parts of the same image. This study investigates the similarity between adjacent rows and columns of a plain image and an encrypted image. The correlation value of the plain image is usually high because there is a strong relationship between adjacent pixels of the plain image. Conversely, the correlation value of the encrypted image is low because strong encryption breaks the relationship of adjacent pixels in the encrypted image. Fig 10 illustrates the correlation relationship between adjacent rows and adjacent columns of the plain and encrypted images.

The figure confirms a strong relationship between adjacent rows and pixels in the plain image. Additionally, it confirms that the proposed image encryption technique successfully disrupts the relationship between adjacent rows and adjacent columns pixels in the encrypted image. The correlation value is computed using the following formula:


$$v_{x}=\frac{\sum_{i=1}^{N}px_{i}}{N},andv_{y}=\frac{\sum_{i=0}^{N}px_{i+1}}{N}$$ (1)

$$d_{x}=\frac{\sum_{i=1}^{N}\left({\left(px_{i}-v_{x}\right)}^{2}\right)}{N},\text{and }d_{y}=\frac{\sum_{i=1}^{N}\left({\left(px_{i+1}-v_{y}\right)}^{2}\right)}{N}$$ (2)

$$Neg_{x_{i},x_{i+1}}=\frac{\sum_{i=1}^{N}\left(x_{i}-v_{x}\right)\left(x_{i+1}-v_{y}\right)}{N}$$ (3)

$$correlation=\frac{Neg_{x_{i},x_{i+1}}}{v_{x}\times v_{y}}$$ (4)

**Fig 11:**
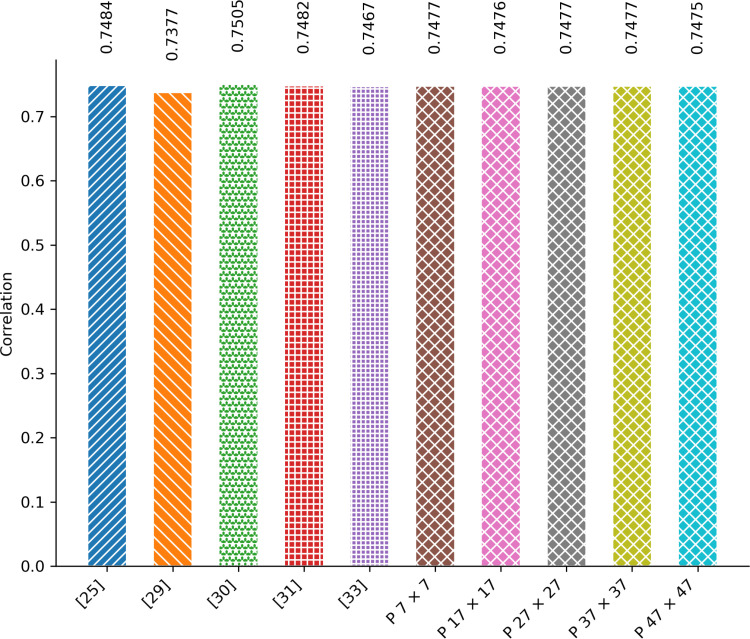
Correlation Comparison.

Fig 11 presents a comparison of the correlation between the proposed scheme and existing image encryption schemes. The results are based on the average correlation value across the entire dataset. For the plain dataset, the average correlation value is 0.9948, indicating a strong relationship between adjacent pixels in the plain images. However, Fig 11 demonstrates that all image encryption schemes notably decrease the correlation between adjacent pixels in both rows and columns of the encrypted images. Furthermore, there’s no significant difference observed in the correlation value when comparing the proposed technique with the existing image encryption technique.

### 4.5. Mean Square Error (MSE)

MSE serves as a metric to quantify the average squared difference between pixel values in two images. It is highly beneficial in assessing the impact of encryption algorithms by comparing an original image with its encrypted counterpart. A higher MSE value indicates greater alteration between the original and encrypted images, which can imply enhanced security against attacks—an advantageous characteristic in encryption algorithms. MSE is computed using the following formula:


$$Mean\_Square\_Error=\frac{1}{N\times M}\left(\overset{M}{\underset{i=1}{\sum }}\overset{N}{\underset{j=1}{\sum }}{\left(I_{plan}\left(i,j\right)-I_{encrypted}\left(i,j\right)\right)}^{2}\right)$$ (5)

**Fig 12:**
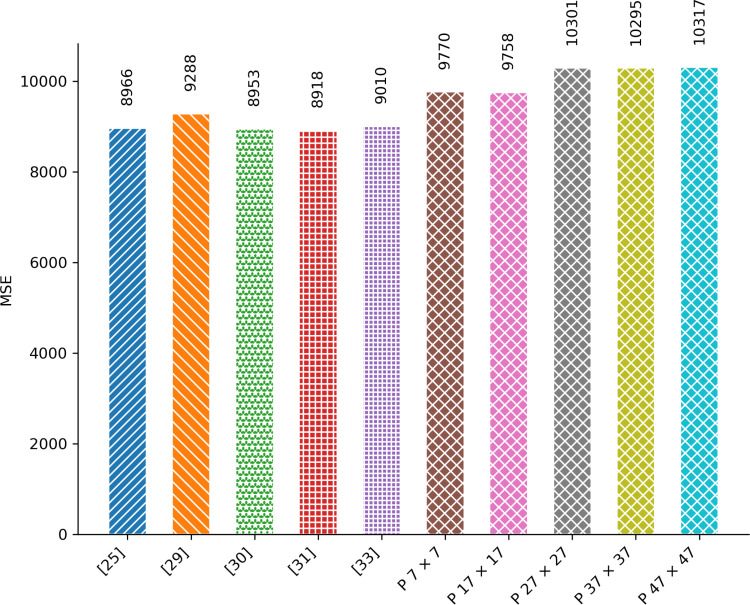
MSE Comparison.

The MSE comparison of the proposed and existing image encryption techniques is shown in Fig 12.

The graph illustrates that the MSE value of the proposed image encryption technique outperforms that of existing state-of-the-art methods. Additionally, it reveals a progressive rise in the MSE value of the proposed image encryption algorithm as the tile size increases.

### 4.6. Security analysis

Key space Analysis: The proposed image encryption technique relies on two values that require protection: (a) a chaotic sequence X and (b) the 512-bits hash of the original image. To generate the chaotic sequence X, two initial values, x and r, are needed. The value of x must be within the range of 0 to 1, while the value of r should fall between 3.57 and 4.0. Notably, x for the logistic map contains 15 varied digits in its decimal representation, and the parameter r has 16 decimal places. In total, three values can be used as keys in the proposed image encryption technique: (a) the hash of the original image, (b) the value of x, and (c) the value of r. Therefore, the total key space can be calculated as follows:


$$KeySpace=10^{15}\times 10^{16}\times 2^{512}$$

This analysis confirms that the key space is sufficient to provide protection against brute-force attacks.

The proposed image encryption technique uses various operations, such as variable tile sizes, chaotic shuffling, random key generation, XOR operations, hashing, non-repetitive key generation, and a random initialization vector to provide protection against various attacks, such as known plaintext attacks, known ciphertext attacks, chosen plaintext attacks, and chosen ciphertext attacks. The in-depth analysis of the proposed scheme is given below:

**Known Plaintext Attack:** The proposed image encryption technique partitions the image into tiles of varying dimensions. This non-uniformity makes it challenging to identify the patterns between the plain image and the cipher image. Secondly, the chaotic shuffling of tiles based on sensitive initial parameters makes it difficult to deduce the exact relation between them. Finally, the generation of the key sequence X based on a hash of the original image and additional random values makes it difficult for the attacker to predict the key based solely on the plaintext.

**Known Ciphertext Attack**: The proposed technique encrypts each tile by applying multiple XOR operations on the plain tile, a hash of the original image, and the IV. Moreover, the value of the IV is updated after the encryption of each tile. This complex relation makes it difficult for attackers to retrieve the plain image from the cipher image. Moreover, the use of the hash value in the encryption process and the generation of the key ensures that a small change in the plain image will generate a completely different cipher image. Furthermore, the involvement of a unique hash generates a non-repetitive key for each image. If two plain images are encrypted using the proposed technique, a completely different cipher image will be generated due to the involvement of unique key sequences.

**Chosen Plaintext Attack:** The IV is XORed with the pixels of each tile, adding an extra layer of randomness as the IV value is updated after each iteration. Even if the attacker chooses a plain image to encrypt, the resulting cipher image will vary based on the IV. Secondly, the key and shuffling pattern are generated from the hash of the original image and other random values. Hence, it is not possible for attackers to predict the output, even if the attacker is able to choose plaintexts. Lastly, the tiles are shuffled based on a random chaotic sequence before the encryption. This setup hides the relationship between the chosen plain image and its cipher image.

**Chosen Ciphertext Attack:** The decryption process uses the same complex steps to decrypt the image. The ciphertext is carefully managed so that it does not provide any insights into the encryption parameters. Secondly, the chaotic reshuffling of tiles to their original positions based on sensitive initial parameters makes it difficult to deduce the exact relation between them.

**Differential Attack:** In this type of attack, the attacker observes the difference between a pair of plain images and their corresponding cipher images to get the secret key or break the encryption algorithm. The proposed technique is designed with several protective measures, such as a large key size, dynamic key generation, chaotic systems, multiple XOR operations, and random IVs to provide protection against differential attacks.

It can be concluded from the above discussion that if the attacker can influence the inputs or observe outputs, even then, it is challenging for the attacker to extract useful information.

### 4.7. Sensitivity analysis

One of the essential properties of an encryption algorithm is the avalanche effect, where a small change in the plaintext image should cause a significant change in the encrypted image, affecting approximately half of the pixels. The sensitivity analysis helps us determine whether the proposed encryption algorithm achieves the avalanche effect. It assesses two key metrics: the Number of Changing Pixel Rate (NPRC) and the Unified Averaged Changed Intensity (UACI). Ideally, NPRC values should exceed 99, while UACI values should be greater than 33 for a secure image encryption scheme. These metrics provide insights into how effectively the algorithm disperses changes throughout the encrypted image, which is crucial for robust encryption techniques. Table 4 compares the proposed image encryption technique with existing image encryption techniques. The results demonstrate that the proposed technique achieves the desired property more effectively than the existing image encryption techniques.

**Table 4:** UACI and NPRC Comparison.

	[25]	[29]	[30]	[31]	[33]	P 7 by 7
**UACI**	33.45712472	0.000217394	2.83977979	0.00021	33.48048571	34.23375269
**NPRC**	99.95088577	0.000412634	93.02949945	0.000413	99.36499812	99.4161517

### 4.8. Striking a balance: lightweight design vs. security

While designing the proposed image encryption scheme, we prioritized keeping the core encryption operation as lightweight as possible, which is why the core operation relies on XOR across multiple attributes. We conducted various analyses to reduce the number of attributes used in the scheme. We reached the conclusion that further reducing the number of attributes would improve the overall encryption/decryption time but at the cost of compromising privacy. Our observations indicated that patterns become visible in the encrypted images when the number of attributes is further decreased. The experimental results also confirm that the proposed image encryption technique provides better performance than most of the existing state-of-the-art image encryption techniques. Secondly, we have divided the image into multiple tiles. Increasing the number of tiles leads to longer encryption and decryption times; however, the resulting ciphertext exhibits enhanced confusion and diffusion properties. Therefore, the choice of tile size can be tailored based on the device’s capabilities and security requirements. Lastly, the NIST randomness test, sensitivity analysis, and security analysis confirm that the proposed scheme is secure against known attacks, thereby providing strong security.

In summary, the image encryption scheme discussed in [25] successfully passed all NIST randomness tests and demonstrated satisfactory performance across various parameters, except for encryption and decryption times, which are approximately seven times longer compared to other schemes examined in the experimental study. Similarly, the schemes discussed in [29–31] are lightweight compared to the scheme presented in [25]. However, those schemes failed multiple NIST randomness tests, and sensitivity analysis also indicates their lack of security. The scheme presented in [33] is lighter compared to [25], with acceptable sensitivity analysis values, but it failed two NIST randomness tests. In overall comparison, the proposed image encryption scheme outperforms all existing schemes. The encryption/decryption time is significantly improved than [25], demonstrates confirmed security through sensitivity analysis, and successfully passes all NIST randomness tests.

## 5. Conclusion

A variety of IoTs applications across different domains rely on transmitting image data and require robust protection measures, such as smart home security cameras, traffic monitoring systems, healthcare monitoring systems, agricultural monitoring systems, autonomous vehicles, remote sensing, and environmental monitoring. The limited processing and storage capabilities of IoT devices make them unsuitable for the implementation of conventional encryption methods. There is always a need for lightweight image encryption techniques that provide a sufficient level of protection within limited processing power for such resource-constrained environments. Most of the existing solutions fail to provide sufficient security within limited processing power. Our proposed scheme not only provides sufficient security but also operates efficiently within the constraints of limited processing power.

Although the proposed technique achieves desirable performance and security metrics, there is potential for improvement in the encryption/decryption time. Our future plans involve enhancing this while ensuring it continues to pass all NIST randomness tests.
